# Cortical brain activity in transfemoral or knee-disarticulation prosthesis users performing single- and dual-task walking activities

**DOI:** 10.1177/2055668320964109

**Published:** 2020-11-05

**Authors:** Saffran Möller, Nerrolyn Ramstrand, Kerstin Hagberg, David Rusaw

**Affiliations:** 1 School of Health and Welfare, Jönköping University, Jönköping, Sweden; 2 ADULT Research Group, Jönköping University, Jönköping, Sweden; 3 CHILD Research Group, Jönköping University, Jönköping, Sweden; 4 Advanced Reconstruction of Extremities, Sahlgrenska University Hospital, Gothenburg, Sweden; 5 Department of Orthopaedics, Institute of Clinical Sciences, Sahlgrenska Academy, University of Gothenburg, Sahlgrenska University Hospital, Gothenburg, Sweden

**Keywords:** Functional near-infrared spectroscopy, prosthetic limb, cognitive load, brain activity, amputee

## Abstract

**Introduction:**

Walking with a prosthesis while performing secondary tasks increases demand on cognitive resources, compromising balance and gait. This study investigated effects of a secondary task on patterns of brain activity and temporospatial gait parameters in individuals using a prosthesis with or without a microprocessor-controlled prosthetic knee(MPK) and controls.

**Methods:**

A cross-sectional study with repeated measures was performed. Twenty-nine individuals with amputations and 16 controls were recruited. Functional near-infrared spectroscopy was used to evaluate changes in oxygenated and de-oxygenated haemoglobin in the prefrontal cortex and temporospatial variables during single-and dual-task walking.

**Results:**

Differences in brain activity were observed within the MPK-group and controls without changes in temporospatial parameters. The Trail-Walking test was associated with highest levels of brain activity in both groups. No differences were observed between single- and dual-task walking in the non-MPK-group (p > 0.05). The Non-MPK and the MPK-group recorded higher levels of brain activity than controls during single-task walking and poorer results on temporospatial variables compared to controls.

**Conclusions:**

For the MPK-group and controls, introduction of a secondary task led to an increase in brain activity. This was not seen in the Non-MPK-group. Significant differences in brain activity were observed in the absence of changes in temporospatial parameters.

## Introduction

When walking, the ability to perform two tasks simultaneously, dual-tasking, is an important skill. It allows us to respond to unexpected events - a bump or threat to our balance - or to do two things simultaneously, such as walk and pay attention to traffic.^1^ Research has shown that a decreased ability to dual-task increases the risk of falls^2,3^ and negatively affects walking performance.^4^ One theoretical explanation for this is provided by Kahneman^5^ who suggests that the human brain has a limited capacity for processing information and that, when attentional capacity is reached, task performance becomes compromised. In the case of dual-tasking, this can mean that one task needs to be prioritized over the other^5^ (e.g. focusing on walking rather than talking).

For people using a lower-limb prosthesis, performance of a secondary task while walking has been shown to affect balance and gait negatively.^6^ This may offer one explanation why prosthesis users fall more frequently and experience an increased fear of falling.^7^ There is some suggestion that prescription of microprocessor-controlled prosthetic knees (MPK) may help to reduce cognitive load during walking and subsequently reduce the risk of falls and balance disturbances.^8^ These knee joints utilize adaptive, computerized systems which continuously adjust the mechanical properties of the knee to adapt to the user’s needs in varying conditions (e.g. altering speed, compensating for a stumble or adapting to varying terrain).^9^ When compared to walking with a non-MPK, walking with an MPK has been shown to increase balance confidence, improve the ability to walk on uneven terrain, reduce the attentional demand required to walk and reduce falls.^9^ Recently, Moller et al.^8^ reported reduced activity in the frontal cortex during single-task walking of individuals using an MPK compared to those using a non-microprocessor-controlled prosthetic knee (non-MPK). There is however limited knowledge of the effects that dual-tasking may have on brain activity, or if choice of prosthetic components can influence levels of activity in the brain when secondary tasks are performed.

One method which is gaining popularity for investigating cortical brain activity in dynamic situations is functional near-infrared spectroscopy (fNIRS).^10^ This method records relative changes in oxygenated and deoxygenated blood flow to specific regions of the brain. In the case dual-tasking, the prefrontal cortex of the brain is a key area of interest as it is associated with attentional demand. Recent research using fNIRS has demonstrated that cortical activity in the prefrontal cortex of the brain increases when young and elderly adults perform a secondary task while walking.^10,11^

## Aim

The aim of this research was to study effects of single- and dual-task walking on cortical brain activity and temporospatial gait parameters in individuals using a non-MPK, MPK and a group of controls. A second aim was to investigate differences in cortical brain activity and temporospatial gait parameters between groups of individuals using a non-MPK, an MPK and controls.

## Methods

### Participants

A cross-sectional, repeated-measures design was used. Twenty-nine individuals with unilateral transfemoral (TF) or knee-disarticulation (KD) amputations were recruited from prosthetic and orthotic clinics in Sweden and Norway. Participants were required to be 18 years or over without any additional physical limitations, able to walk continuously with their prosthesis for 500 meters using a maximum of one gait aid, i.e. crutch or walking stick and able to understand written and spoken Swedish or Norwegian. A group of controls matched by age and sex (n = 16) were also recruited from the community. Individuals with a bone-anchored prosthesis or cognitive impairment (Mini Mental State Examination test <27) were excluded.^12^ Study information and written informed consent was provided prior to testing. Ethical considerations in this research followed the Declaration of Helsinki.^13^ Ethical approval was obtained from appropriate authorities in both Sweden (Linköping, Dnr 2015/215-31) and Norway (Dnr 2015/1526/REK sor-ost).

### Procedure

Prior to data collection, participant characteristics including time since amputation, amputation cause and Prosthetic Use Score^14^ were collected and the testing procedure was demonstrated. Cognitive load was estimated by recording the cerebral haemodynamic response in the brain using an fNIRS system (explained below). The haemodynamic response was measured under three conditions. The first condition was single-task walking (ST) performed on a 14 m, stable, level walkway, while conditions 2 and 3 involved dual-task tests. Condition 2 involved walking on the same 14 m walkway while sorting through keys (KEY). This test was selected to simulate a real world activity and has been used to investigate differences in dual-task performance between prosthetic users and controls.^15^ During this test, participants were requested to hold a keyring comprised of 8 keys (3 different colours and marked with 3 different numbers) in one hand. Participants were then required to begin walking while they tried to identify the appropriate key using just one hand. Condition 3 required that participants complete a modified Trail-Walking Test (TWT), which involved walking while circling a series of numbered cones that had been placed in a 1x4m area in a pre-determined, non-consecutive order. The TWT has been shown to predict increased risk of falling in healthy, elderly individuals.^16^ The area for the modified TWT was less than was used by Yamada & Ichihashi.^16^ This was necessary to accommodate for limited space available in the clinics where testing was performed. Throughout all conditions, participants were instructed to walk at their self-selected velocity. Each condition was repeated four times with the order of numbered cones altered on each occasion.

All testing involving fNIRS was performed in quiet rooms which were free from distractions. Before each testing sequence, a 30-second baseline measurement was recorded while participants were sitting quietly with their eyes closed. This was followed by a period of quiet standing with their eyes open before the walking trial began. In addition to fNIRS data, researchers also recorded the number of steps, time taken to walk 10 m on the walkway (ST and KEY conditions) and time to complete the modified TWT. NIRStar (NIRx Medical Technologies LLC, NY, USA) software was used to collect haemodynamic data while NIRStim software (NIRx Medical Technologies LLC, NY, USA) was used to standardize the testing protocol using pre-recorded verbal prompts and marking time points in the data files. When fNIRS data collection was complete, the apparatus was removed, and participants were requested to complete a six-minute walk test (6MWT). The 6MWT has been shown to distinguish between functional levels in prosthesis users.^17^ Due to limited space the 6MWT was conducted on a 20-meter track rather than the recommended 30 meters. Participants used their normal walking aid for all tests.

### fNIRS data acquisition

A wireless, portable, continuous wave fNIRS system was used (NIRSport tandem (NIRx Medical Technologies LLC, NY, USA)). The system was comprised of 40 optical channels derived from 16 source probes and 16 detector probes. Penetration-depth was approximately 3 cm and a distance of 3 cm was maintained between each source/detector pair. Each probe emitted infrared light at wavelengths of 760 and 850 nm, at a frequency of 7.81 Hz. Optodes were placed over the prefrontal and motor cortices according to a standard montage template (NIRx Medical Technologies LLC, NY, USA). A cap provided a consistent frame of reference for positioning optical probes according to the International 10-20 System.^18^

### fNIRS data processing

Post-processing of fNIRS data was performed using NirsLAB 2017.06 software (NIRx Medical Technologies LLC, NY, USA). While each test condition (ST, KEY, modified TWT) included 4 repetitions, the first sequence was considered a practice trial and removed from the final analysis. For the remaining 3 repetitions, a 10-second period was extracted for analysis beginning 5 seconds after the instruction to walk was given.

All data files were visually inspected. Since no consensus exists regarding removal of bad channels from fNIRS data,^19^ recommendations from the manufacturer were used whereby channels with a gain factor greater than 3 or a coefficient of variation greater than 7.5% were considered as bad channels and removed from analysis. To eliminate signal fluctuations related to factors such as heartbeat, respiration and low-frequency signal drift, a bandpass filter was applied to all data (0.01 to 0.2 Hz).

**Figure 1.**
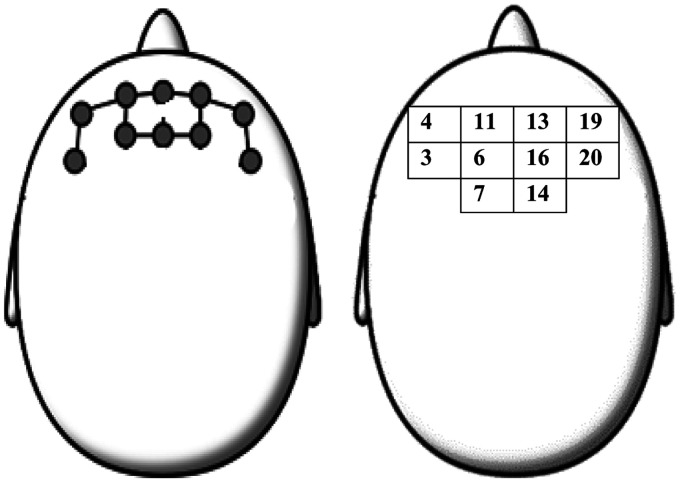
Region of interest (ROI). Circles indicate probes and the lines in-between indicate channels. Numbers indicate channel designation and position.

Relative changes in oxyHb and de-oxyHb were converted to concentrations using the modified Beer-Lambert Law^20^ and concentration changes were normalised relative to baseline values. A region of interest (ROI) representing the left and right prefrontal cortex was identified comprising channels 3, 4, 6, 7 and 11 on the left side and 13, 14, 16, 19 and 20 on the right side (Figure 1). These locations roughly targeted left and right Brodmann's area 10, which is the anterior part of prefrontal cortex and associated with executive functions during dual-task walking.^11^ Time series data for each of the three 10-second trials in each condition were block averaged for each participant using the signals from the region of interest.

### Statistical analysis

IBM SPSS Statistics 21 (SPSS Inc., Chicago, IL) was used for all statistical analyses and the critical alpha level was set at 0.05, unless otherwise stated. Signals were averaged over the 10 seconds test period in accordance with methods earlier described.^21^ Signal fluctuations over time were analysed descriptively by graphing data for the region of interest. The assumption of normality was violated, therefore non-parametric tests were used. To establish if significant differences existed within groups for single-versus dual-tasks, a Friedman's test or Wilcoxon signed-rank test was used. Gait parameters were normalized to height since leg length was not recorded.^22^ Differences between groups (controls, MPK and non-MPK) were evaluated using a Kruskal-Wallis test. When significance was indicated, a post-hoc test with pairwise comparisons was applied. Bonferroni corrections were made to account for multiple comparisons. A Mann-Whitney U test was utilized for comparisons involving only two groups.

## Results

Details of the participants are presented in Table 1. There were no significant differences between the three groups in relation to age, sex or height. Nor were there any differences between the two groups using a non-MPK versus an MPK regarding time since amputation or amount of prosthetic use per week (p > .05). Two participants in the non-MPK-group and two in the MPK-group used one gait aid during the test session. Controls walked a significantly longer distance in the 6MWT compared to individuals using a prosthesis (p < .001) and the MPK-group walked a significantly longer distance than the non-MPK-group (Table 1). Two fNIRS data collection trials were excluded due to technical errors, one involved an ST trial (MPK-group) and one involved a modified TWT trial (non-MPK-group). Two participants did not perform the 6MWT (one due to fatigue and one due to lack of time). Both of these individuals were in the MPK group.

**Table 1.** Participant details.

	Non-MPK (n = 14)	MPK (n = 15)	Controls (n = 16)
	Mean (95% CI)	Mean (95% CI)	Mean (95% CI)
Age years	51 (42–60)	51 (45–57)	47 (40–54)
Female/male	2/12	4/11	5/11
Time since amputation years	19 (11–27)	18 (9–27)	na
Amputation level TF/KD	12/2	11/4	na
Height (cm)	175 (171–178)	176 (172–180)	174 (169–181)
Prosthetic Use Score (0–100)	74 (53–95)	85 (75–95)	na
Six-minute walk test (m)	374** (269–479)	460^a,^* (394–526)	634^◊^ (599–670)

TF = transfemoral amputation, KD = knee-disarticulation amputation. Non-MPK = non-microprocessor-controlled prosthetic knee, MPK = microprocessor-controlled prosthetic knee. In six-minute walk test, two participants were missing in the MPK-group.^a^Two participants are missing.

*Significantly different compared to MPK (p = .033).

^**^Significantly different compared to controls (p = .005).

^◊^Significantly different compared to non-MPK (p < .001).

**Figure 2.**
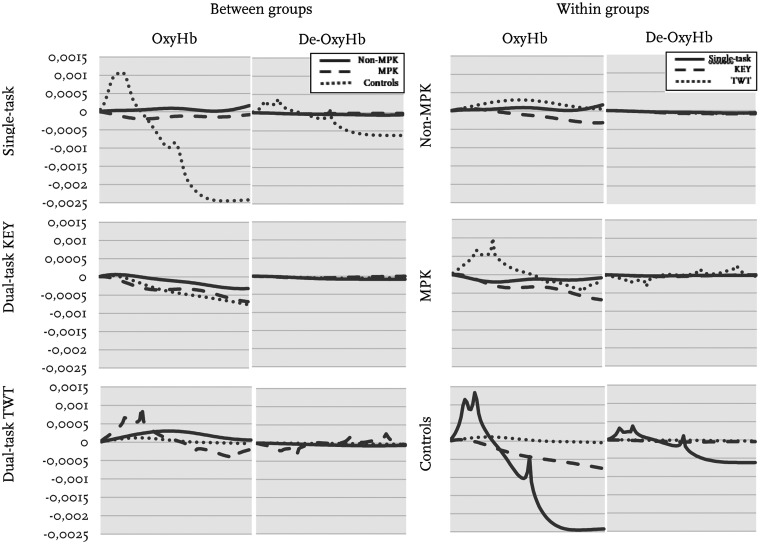
Graphs of the ten seconds period of Mean haemodynamic response in oxyHb and de-oxyHb(mM). To the left single-task and dual-task walking between groups, non-MPK, MPK and Controls are illustrated. To the right within groups non-MPK, MPK and control between single-task, dual-task KEY and dual-task TWT are illustrated.

A Friedman's test revealed a significant difference in oxyHb between the three conditions (ST, KEY and modified TWT) within the group of controls (p = .008) and MPK-group (p < .001). Post-hoc analysis showed that the group of controls had significantly higher mean oxyHb concentrations in the modified TWT compared to the Key test, while the MPK-group had significantly higher mean oxyHb concentration in the modified TWT compared to the single-task walking and in the modified TWT when compared to KEY test (Table 2). This can also be visualized in Figure 2 which presents a graph of oxyHb and de-oxyHb over time. No significant differences were observed in the non-MPK-group between any of the three conditions (ST, KEY and modified TWT). There were no significant differences observed in de-oxyHb concentration changes when comparing the 3 conditions (ST, KEY and modified TWT) in any of the 3 groups (non-MPK, MPK or Controls). Nor were significant differences seen in temporospatial data between single-task and dual-task walking (KEY) within any of the 3 groups, non-MPK, MPK or Controls (p > .05).

**Table 2.** Median of oxyHb and de-oxyHb concentration level changes (mM) in region of interest during the 3 different walking conditions (ST, KEY, modified TWT).

	Single-task walking	Sorting through keys test	Modified trail-walking test	Within group comparisons (post hoc result)
	Median (IQR)	Median (IQR)	Median (IQR)	
Non-MPK				
OxyHb	6.08e5 (6.07e4)	–2.18e4 (8.67e4)	2.46e4 (3.21e4)	p > 0.05
De-oxyHb	–4.86e5 (8.55e5)	–4.24e5 (5.57e5)	–3.30e5 (4.81e5)	p > 0.05
MPK				
OxyHb	0.19e6 (2.93e4)	–4.16e4 (6.91e4)	1.91e4 (5.00e4)	p < 0.001 TWT > ST*, TWT > key**
De-oxyHb	–1.36e5 (3.08e5)	–2.54e5 (6.99e5)	–2.61e5 (4.23e5)	p > 0.05
Controls				
OxyHb	–2.48e4 (4.46e4)	–3.40e4 (2.30e4)	3.27e5 (5.39e4)	p = 0.008 TWT > keys**
De-oxyHb	–1.11e5 (8,45e5)	–4.15e5 (5.34e5)	–1.21e5 (2.44e5)	p > 0.05
Between-group				
OxyHb	p < 0.05^#^ controls > non-MPK& MPK	p > 0.05	p > 0.05	
De-oxyHb	p > 0.05	p > 0.05	p > 0.05	

Non-MPK = non-microprocessor-controlled prosthetic knee joint, MPK = microprocessor-controlled prosthetic knee joint. IQR =  Interquartile Range.

*Single-task walking significantly different from modified TWT (p = .032).

**KEY test significantly different from modified TWT (MPK p<.001, Controls p = 0.007).

^#^ MPK and Non-MPK groups recorded significantly higher values that the control group (non-MPK p = 0.029, MPK p = 0.007).

Between-group comparisons showed a significantly higher mean oxyHb concentration change in both the non-MPK-group (p = .007) and the MPK-group (p = .029) as compared to controls during single-task walking (Figure 2). No other significant differences in haemodynamic response were observed between the groups.

There was no significant difference observed within groups when comparing temporospatial data between single task walking and the key test (p > 0.05). Note that temporospatial data for the modified TWT could not be included in within group comparisons as this test required participants to walk around cones and the walking path was not comparable to that used in the other two tests. Between-group (non-MPK, MPK and Controls) comparisons of temporospatial data showed a significantly reduced cadence in the non-MPK-group as compared to controls and the MPK-group in both the ST and KEY tests. The non-MPK group also took a longer time to complete the modified TWT and walked a shorter distance in the 6MWT. The MPK-group showed a significantly reduced cadence in single-task walking and required a longer time to complete the modified TWT when compared to controls (Table 3).

**Table 3.** Temporospatial data, between-group comparisons for the three walking conditions, single-task walking, sorting through keys (KEY) and modified trail-walking test (TWT).

				Comparison between groups		
	Non-MPK	MPK	Controls	Non-MPK vs controls	MPK vs controls	Non-MPK vs MPK
	Median (IQR) Mean (Std)	Median (IQR) Mean (Std)	Median (IQR) Mean (Std)	P	P	P
Cadence single-task walking	91 (22.61) 95 (11.58)	101 (15.99) 105 (8.46)	112 (14.83) 112 (10.58)	**.001**	**.041**	**.014**
Cadence KEY	96 (17.98) 97 (8.85)	105 (11.51) 106 (7.61)	110 (10.31) 110 (7.22)	**.000**	.188	**.008**
Modified TWT (seconds)	33 (7.42) 33 (5.11)	27 (6.67) 27 (4.06)	21 (4.78) 22 (3.57)	**.000**	**.003**	**.001**

Bold p-values indicate statistical significance. Non-MPK=non-microprocessor-controlled knee, MPK=microprocessor-controlled knee, IQR= Interquartile Range.

## Discussion

The primary aim of this research was to study the effects of single- and dual-task walking on cortical brain activity and temporospatial gait parameters in individuals with amputations using a non-MPK or an MPK and controls. A secondary aim was to investigate differences between groups of individuals using either a non-MPK, an MPK and the control group.

Results revealed an increase in cognitive load (oxyHb) for dual-task walking compared to single-task walking in the MPK group. When compared to the KEY test, the TWT was associated increased activity in the frontal cortex for both the MPK and control groups, suggesting that this test is more cognitively intensive. These findings are consistent with literature concerning healthy young and elderly adults, which has demonstrated that, as gait tasks become more challenging, there is an increased demand on executive functions^10,23^ and increased prefrontal cortex activation.^24^ Decreased performance in dual-tasking has previously been shown to expose a person to higher risk of falls and fall-related accidents.^2,3^ Unfortunately, our study did not include reports of fall history for participants. This would be important to include in future research.

Irrespective of activity, an increase in oxyHb concentration is typically followed by a decrease in de-oxyHb. This was not seen in this study and no significant differences were observed in de-oxyHb. Some authors suggest that oxyHb is the most sensitive measurement^25^ although de-oxyHb is sometimes reported to be the more stable variable.^26^

In the group using a non-MPK there were no significant differences in oxyHb or de-oxyHb between single-task and dual-task walking. This was an unexpected result as previous research has demonstrated a clear association between increased concentration of oxyHb and increased cognitive load during dual-task walking.^10,11^ According to Kahneman’s Capacity Theory^5^ it is possible that the non-MPK-group reached their maximum attentional capacity during single-task walking and that no additional increase was possible. If this were the case, one would expect that gait performance, or performance on the secondary task, would be compromised.^27^ In the present study, performance of the secondary task was not evaluated while gait performance was assessed using temporospatial measures with no significant differences observed when comparing single-task and dual-task walking. This is noteworthy, as it suggests that elements of gait performance that can be observed with ease in the clinical setting may not be affected by the addition of an attentionally demanding task, even when there are measurable differences in the haemodynamic response. This has clinical implications as the relative effects of attentionally demanding tasks may not be observable during routine gait analysis. Results support findings of previous authors who have suggested that assessments of executive functioning, such as dual-task coordination, should be used to a greater extent in the rehabilitation setting.^28,29^

Between-group analyses revealed a significant difference in haemodynamic response in ST walking where both groups with prostheses recorded a higher mean oxyHb compared to controls. This is consistent with earlier research showing that older adults and individuals walking with a prosthesis have increased cognitive load during walking.^3,8^

Between-group analyses of temporospatial data revealed that individuals using a non-MPK walked significantly slower, with a reduced cadence, a reduced distance in 6MWT and took a longer time to complete the modified TWT compared to both controls and those who used an MPK. Given that MPK-joints are typically prescribed for individuals with a high functional level, it is possible that results reflect baseline differences even though there were no significant differences between the non-MPK and MPK-groups in Prosthetic Use Scores, age and time since amputation.

### Study limitations

This study has some methodological limitations worth discussing. The three test conditions were not randomized. As such, the order of walking conditions cannot be excluded as a confounding factor. In addition, brain activity may have been influenced by the characteristics of the secondary task. The secondary tasks used in this study involved combined cognitive and motor activities. In the modified TWT participants hade to identify, and maneuver around, numbered cones while the KEY test required them to find a specific key. It would be of interest to investigate secondary tasks that are purely cognitive in nature e.g., verbal fluency. While the fNIRS system offers the advantage of portability, results can be affected by noise such as motion artefacts, probe placement, skull thickness or skin response.^30^ Although every attempt was made to address the relative effects of noise, we cannot with absolute certainty exclude its effects on the results in this study.

Differences in physical- and amputation-related factors (e.g. residual limb length, strength), and prosthetic related factors (e.g. type of socket, alignment, foot component) may also have had an influence on the results. These issues require further investigation. Finally, the relatively small and homogenous sample size means that result cannot be generalized to all individuals who use a transfemoral or knee-disarticulation prosthesis.

## Conclusions

The current findings indicate that addition of a secondary task during walking increases cortical brain activity in the prefrontal cortex in adults who have had an amputation and walk with an MPK. The trail-walking test induced a greater cortical response than a task requiring sorting through keys (MKP group and controls). Within group differences in brain activity were not observed in temporospatial measures. This can be of importance for clinicians who routinely evaluate gait and suggests that more attention should be given to the relative effects of cognitive load on walking performance.

Greater cortical brain activity was observed in single-task walking for individuals using a prosthesis (non-MPK and MPK) compared to a control group of people who did not use a prosthesis.
